# Peripartum Cardiomyopathy in Intensive Care Unit: An Update

**DOI:** 10.3389/fmed.2015.00082

**Published:** 2015-11-23

**Authors:** Vesna Dinic, Danica Markovic, Nenad Savic, Marija Kutlesic, Radmilo J. Jankovic

**Affiliations:** ^1^Center for Anesthesiology and Reanimatology, Clinical Center of Nis, Nis, Serbia; ^2^Department for Anesthesiology and Intensive Care, School of Medicine, University of Nis, Nis, Serbia

**Keywords:** peripartum cardiomyopathy, heart failure, pregnancy, monitoring, treatment

## Abstract

Peripartum cardiomyopathy (PPCM) is a systolic heart failure that occurs during the last month of pregnancy or within 5 months after delivery. It is an uncommon disease of unknown etiopathogenesis and has a very high rate of maternal mortality. Because of similarity between symptoms of PPCM and physiological discomforts during pregnancy, the early diagnosis of PPCM presents a major challenge. Since hemodynamic changes during PPCM can vitally jeopardize the mother and the fetus, patients with severe forms of PPCM require a multidisciplinary approach in intensive care units. This review summarizes the current state of knowledge about the diagnosis, monitoring, and the treatment of PPCM. Having reviewed the recent researches, it gives insight into the new treatment strategies of this rare disease.

## Introduction

Even at the beginning of the twenty-first century, heart diseases continue to be the leading cause of maternal deaths in many countries (1). One of the most severe heart diseases related to pregnancy is peripartum cardiomyopathy (PPCM).

Peripartum cardiomyopathy is a rare and very serious type of idiopathic cardiomyopathy, presenting with a left ventricular systolic dysfunction and heart failure during the last month of pregnancy or up to 5 months after delivery, in previously healthy women (2).

This time limit is very important as an exclusion factor for other previously undiagnosed or preexisting types of cardiomyopathies, which can be unmasked by hemodynamic changes during pregnancy. Therefore, it is very important to have in mind the fact that a woman without a history of a heart disease can develop heart failure in the peripartum period.

For the first time, PPCM was defined in 1971 by Demakis et al. who investigated 27 patients with cardiomegaly, abnormal ECG, and heart failure in the puerperium (3). They defined three diagnostic criteria for PPCM: the development of heart failure during the last month of pregnancy or within 5 months after delivery, the absence of determinable etiology for heart failure, and the absence of heart disease before the last month of pregnancy (3, 4). Recently, echocardiographic diagnostic criteria for the left ventricular dysfunction, such as left ventricular ejection fraction (LVEF) <45%, left ventricular fractional shortening (LVFS) <30% or both, and left ventricular end-diastolic dimension (LVEDD) >2.7 cm/m^2^ body surface area, have been included in the diagnosis of PPCM (5). These criteria made the definition of PPCM more precise and facilitated differentiation between PPCM and other forms of dilated cardiomyopathy (Table 1).

**Table 1:** **Diagnostic criteria for peripartum cardiomyopathy**.

Demakis criteria (3, 4)	Echocardiographic criteria
Heart failure within last month of pregnancy up to 5 months after delivery	LVEF <45%
Absence of determinable cause for the heart failure	LVFS <30%
Absence of heart disease before the last month of pregnancy	LVEDD >2.7 cm/m^2^ body surface area

*LVEF, left ventricular ejection fraction; LVFS, left ventricular fractional shortening; LVEDD, left ventricular end-diastolic dimension*.

## Epidemiology

The incidence of PPCM varies among different countries. Very high incidence (1:299) is reported in Haiti (6), while in South Africa, it is 1:1000 (7) and in the USA, 1:2289–4000 (8). Although it is an uncommon disease, PPCM is associated with very high maternal mortality rate that can reach 28% after 6 months despite the therapy. (9). Those who survive can develop chronic heart failure and most of them require heart transplantation (9). There are numerous risk factors for PPCM such as: advanced maternal age, breastfeeding, multiparity, multifetal pregnancies, pregnancy induced hypertension, preeclampsia, and prolonged use of tocolytic therapy (3, 4, 10). The African-American ethnicity is considered to be a high risk factor for PPCM (11).

## Clinical Presentation

Pregnancy is a physiological state accompanied by maternal hemodynamic changes, during which time, signs and symptoms could mimic signs of heart failure. Awareness of these changes is of great importance for early recognition and timely management of PPCM, which correlate with better prognosis of these patients. Symptoms of PPCM, such as dyspnea, cough, fatigue, leg edema, malaise, are very often misinterpreted as physiological symptoms due to pregnancy. This is the reason of delayed recognition of PPCM, so at the moment of diagnosis patients are presented with NYHA III or IV functional class (12).

The clinical course of PPCM can vary from mild to extremely severe heart failure and even cardiogenic shock, which can vitally jeopardize both the mother and the fetus. Progression to heart failure can be rapid, very often within few days from the first presentation of symptoms. Paroxysmal nocturnal dyspnea, nocturnal cough, hemoptysis, chest pain, and hepatomegaly should raise suspicion of heart failure (13). Tachycardia, murmurs, pulmonary rales, and increased jugular pressure can be revealed at physical examination. Premature ventricular beats, atrial tachycardia, ventricular tachycardia, and cardiac arrest were described in these patients (14, 15). Left ventricular thrombus and thromboembolic events can be present (16), and in the worst clinical scenario, multiorgan failure can occur (17). When considering PPCM, we should always have on mind a possibility of latent forms. A study from Haiti reported four women with PPCM without clinical symptoms, but with echocardiographic findings specific for this disease (18).

## Etiopathogenesis

Despite numerous researches, the etiopathogenesis of PPCM is still unknown. There are many hypothesis including myocarditis (19, 20), genetic succeptibility, fetal microchimerism, and autoimmune response (21), which could be an explanation for higher incidence of PPCM in multiparity (22).

Since the cause of PPCM is unknown, there is still no specific treatment of this serious disease. Understanding of etiopathogenesis could represent a breakthrough in the treatment of PPCM. From that aspect, studies that investigated inflammation, prolactin, and oxidative stress as a possible cause of PPCM reported promising results.

In a large prospective study, Sliwa et al. reported significantly higher levels of inflammatory markers – CRP, IL6, TNFα, and plasma marker of apoptosis (Fas/Apo-1) in patients with PPCM than in healthy controls (23, 24). They found positive correlation between inflammatory markers and LVEDD and LVESD, as well as inverse correlation between markers and LVEF. Significantly high value of Fas/Apo-1, as a marker of cardiomyocyte apoptosis in PPCM patients, was found to be a predictor of mortality. These researches suggest that inflammation could play a role in pathogenesis of PPCM. In that case, inflammation markers could be used in clinical practice as predictors of complications and mortality. Also, these researches are important from the aspect of treatment of PPCM and use of TNF inhibitor as a target therapy.

In contrast to these researches, other studies suggest that PPCM is a consequence of vascular disorders due to cleavage of PRL and increased oxidative stress.

**Figure 1:**
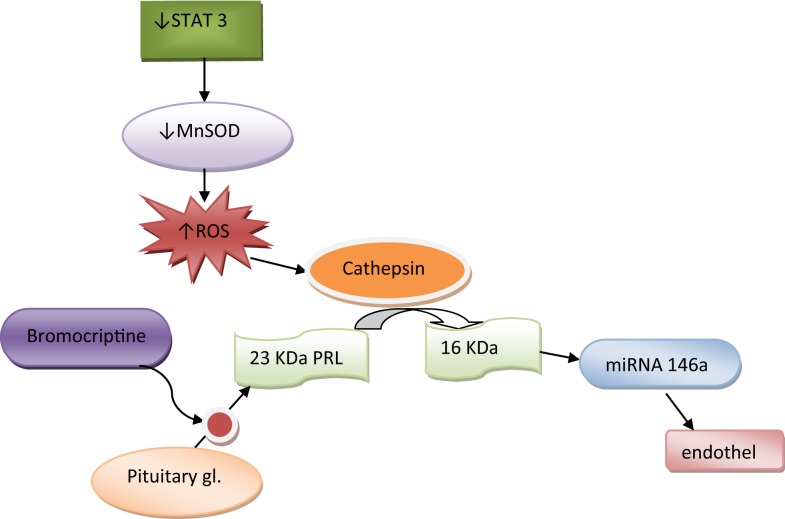
**Pathogenesis of peripartum cardiomyopathy**.

Hilfiker-Kleiner et al. have found that decreased levels of signal transducer and activator of transcription 3 (STAT3) in mice induce decreased activity of manganese superoxide dismutase (MnSOD) leading to increased oxidative stress. Reactive oxygen species (ROS) accumulation induces cathepsin D activation, which further cleaves nursing-hormone prolactin of 23 kDa (23 kDa PRL) into 16 kDa prolactin (16 kDa PRL), which was proved to have antiangiogenic and proapoptotic effects on cardiomyocytes (25). Halkein et al. explained antiangiogenic effects of 16 kDa PRL by expression of micro-RNA146a (26), which attenuates angiogenesis (26). They found high levels of micro-RNA146a only in patients with PPCM, but not in those with dilated cardiomyopathy. They suggested that micro-RNA146a could be used as a biomarker and therapeutic target for PPCM (26). In another study, deficiency of VEGF was found to contribute to PPCM (27). Increased levels of PRL, ROS, and 16 kDa PRL are also found in women with PPCM (25), suggesting that this mechanism could play a central role in the pathogenesis of PPCM. The use of bromocriptin, inhibitor of PRL, removed adverse effects of 16-kDa PRL and prevented PPCM in mice (25). This highlighted the possibility of its use as a new specific drug in the treatment of PPCM (Figure 1).

From the clinical point of view, data from these studies may have an impact on the new treatment strategies of PPCM in the future.

## Diagnosis

When considering PPCM, it is very important to have in mind the fact that a woman without a history of a heart disease can develop heart failure in the peripartum period. Thus, rising awareness of PPCM is very important for early diagnosis. Delayed diagnosis is followed by higher incidence of complications. When the diagnosis is made before LVEF decline below 0.35, the mortality rate approaches 0, and the chance of full recovery is much greater. Because of similarity in clinical presentation between PPCM and other types of systolic heart failure, the diagnosis of PPCM presents a major challenge. It is based on exclusion of other conditions that could cause heart failure: idiopathic dilated cardiomyopathy, myocardial infarction, severe preeclampsia, pulmonary embolism, sepsis, and valvular heart disease. Diagnostic criteria proposed by Demakis (3) are very important as an exclusion factors for preexisting cardiomyopathies. The most important is to make an early diagnose of PPCM. Initial assessment includes: history, detailed physical examination, routine blood analysis, and electrocardiography.

Electrocardiogram patterns are not specific for PPCM. ST-T wave abnormalities, bundle branch block, atrial fibrillation, and ventricular tachycardia can be seen (28), as well as myocardial infarction. Chest X-ray can be done only after delivery, and it can reveal cardiomegaly, pleural effusion, and pulmonary congestion, while MRI can be used to distinguish myocarditis and ischemia as possible causes of PPCM and to estimate segmental and global myocardial contractility.

The definitive diagnosis of PPCM is based on echocardiographic findings. Echocardiography enables prompt diagnosis and the evaluation of heart function. Thus, in any case of doubt or suspicion of heart failure, bed-side echocardiography should be done as soon as possible. PPCM is presented with systolic dysfunction, reduced cardiac output, and increased filling pressures (29), while left ventricular dilatation must not be always present (30). Echocardiographic diagnostic criteria for PPCM include: EF <45%, LVFS <30% or both, and LVEDD >2.7 cm/m^2^ body surface area (5). Valvular regurgitation can be present. In patients with EF ≤35%, left ventricular thrombus are often detected (31). In cases of preeclampsia with heart failure, it is very important to distinguish if it is PPCM in preeclamptic patient or heart failure as a complication of preeclampsia. In that situation, echocardiographic findings will distinguish heart failure with reduced ejection fraction (PPCM) and normal ejection fraction (preeclampsia). Diastolic dysfunction, preserved ejection fraction, left ventricular hypertrophy, and non-dilated ventricles are specific for preeclampsia (29, 32).

Biomarkers could be very helpful in early diagnosis of PPCM. Unfortunately, there are still no specific biomarkers for PPCM. Brain natriuretic peptide (BNP) and N-terminal portion of pro BNP (pro-NT BNP) are biomarkers sensitive for heart failure, but are not specific for PPCM. However, these markers in combination with echocardiography can be very useful for risk stratification and accuracy of diagnosis in patients with asymptomatic PPCM in cases when levels of BNP and pro-NT BNP are intermediate (33). Several new studies reported promising results regarding biomarkers in PPCM. Data from these studies reported significantly higher levels of micro-RNA-146a in patients with PPCM compared to healthy women and women with dilated cardiomyopathy (26, 34), which suggest that micro-RNA-146a could be used as a specific biomarker, and also therapeutic target in patients with PPCM. However, further studies are needed before it can be used as a marker in standard clinical practice. Recent researches in the field of pathogenesis suggest that CRP, Tiff, and IL 6 could be used as predictors of complications and mortality in PPCM (23, 24).

Because of the complexity of this disease, which can be fatal for both mother and the fetus, PPCM should be managed by multidisciplinary team. Patients admitted in the ICU require intensive treatment and hemodynamic monitoring.

## Monitoring

Monitoring before and 24 h after delivery is very important because it is the period of the most prominent hemodynamic changes. Understanding of physiological hemodynamic changes during pregnancy is of great importance for interpretation of hemodynamic parameters of PPCM. Auto transfusion due to uterine contractions and fluid shifts after delivery could alter the condition of patients with PPCM, augment the fluid load, and cause acute heart decomposition. PPCM presents with decreased cardiac output and increased right and left heart filling (35).

The choice of monitoring devices depends on the severity of clinical presentation. Intra-arterial blood pressure should be measured in severe forms of PPCM followed by intense hypotension. Because of the risk of potential complications, central venous catheter and pulmonary artery catheter are not routinely used, except in cases of severe PPCM. However, in a patient with dyspnea at rest, severe chest pain, ECG evidence of myocardial ischemia, severe impairment of myocardial contractility, invasive monitoring with a Swan Gang catheter is justified (36). The use of continuous non-invasive cardiac output monitoring (NICOM™) in patient with PPCM was reported. Since good correlation between cardiac output monitoring with NICOM and with pulmonary artery catheter has been shown, the authors suggest that it can be used as a guide in the treatment of PPCM and also for early detection of heart decomposition (37).

Tran esophageal echocardiography (TEE) is rarely used in obstetrics, because it very often requires tracheal intubation and can be performed only by an experienced practitioner. However, some authors suggest it as a guide in management of refractory hypotension and cardiac arrest in obstetric patients (38), thus TTE could find its place in very severe forms of PPCM.

Tran thoracic echocardiography (TTE) is crucial not only for diagnosis of PPCM but also for evaluation and monitoring of the effects of the therapy. It enables the intensives to get non-invasive serial evaluations of the heart function and structure at the bedside, without risk of complications. PPCM presents with decreased EF, systolic dysfunction, decreased LVFS, decreased cardiac output (5). TTE is the best tool for detecting intracranial thrombi. Cardiac output can be accurately measured using TTE (39).

## Management

The treatment of PPCM does not differ from that of other types of dilated cardiomyopathy, but the choice of medications depends on fetal safety during ante partum heart failure and on excretion of the drug during breastfeeding.

In the cases of acute decompensate heart failure, the initial management is based on ABCs (airway, breathing and circulation) (40). Patients with pulmonary edema often require supported ventilation. Since the non-invasive ventilation is accompanied by the higher risk of aspiration in pregnant women, end tracheal intubation should be considered. Arterial oxygen saturation ≥95% should be achieved (41). Hemodynamic changes during PPCM, such as decreased cardiac output, can alter uteroplacental perfusion and cause fetal distress; thus fetal heart rate monitoring is mandatory. PPCM is not an indication for delivery except in cases of hemodynamic unstable patient or rapidly decompensating patient.

Attention should be paid to the clinical assessment of decreased cardiac output and hypoperfusion rather than on the exact value of blood pressure, which must not always indicate hypotension (42). The treatment of PPCM is based on improvement of hemodynamic status of the patient by decreasing preload and afterload, and by increasing cardiac contractile force (43, 44).

Angiotensin converting enzyme inhibitors (ACEI) efficiently reduce afterload, but due to their proven teratogenic effects they are contraindicated during pregnancy. In antenatal heart failure, vasodilatators such as hidralazine and nitroglycerin can be safely used instead of ACEI and ARB, but should be carefully titrated if systolic blood pressure is between 90 and 110 mm Hg (41). ACEI can be used after delivery. Benazepril, captopril, and enalapril were tested in nursing women and their use has proven to be safe for babies (45). ACEI improve survival of patients with PPCM (46). Patients who cannot tolerate ACEI can be treated with angiotensin-receptor blockers (ARB) (47).

Sodium nitroprusside should be avoided because of thiocyanate toxicity. Loop diuretics reduce preload and fluid overload and relieve symptoms of PPCM, but should be used with caution because they can cause hypotension, uterine hypoperfusion and, consequently, fetal distress.

Digoxin has positive inotropic effects and improves ejection fraction in combination with vasodilatators. It can be safely used during pregnancy and breastfeeding (48). In cases of severe decompensated antenatal heart failure with hypotension, dobutamine and dopamine are the drugs of choice.

The use of levosimendan in PPCM is still controversial. There are case reports about the successful use of levosimendan (49, 50), but randomized controlled trials are needed to prove its positive effects on outcome in PPCM.

Beta blockers are indicated in PPCM, and some authors suggest that they should be continued for at least 1 year (51). Beta-1 blockers are preferred, as they do not interfere with uterine tone, while beta non-selective blockers should be avoided because of anti-tocolytic effects. Alpha and beta blocker, carvedilol, has been shown to be very effective in PPCM (44).

Uterotonics should be very carefully used in PPCM. Oxytocin decreases systemic vascular resistance, compensatory increases heart rate, and can cause coronary vasoconstriction, so is poorly tolerated in state of LV dysfunction. Therefore, it should be slowly titrated. Ergometrine causes coronary and pulmonary vasoconstriction, and should be avoided in PPCM (52). Postpartum analgesia is very important in patients with PPCM. Pain-induced sympathetic activation leads to tachycardia and increase in afterload, which further compromise heart function. Therefore, epidural analgesia is preferred for labor, and it can be continued in the ICU. Thromboembolic events are common in PPCM. It is recommended that patients with LVEF <35% should receive anticoagulation therapy (53).

Arrhythmias are not rare in PPCM. Refractory life-threatening arrhythmias in patients with poor left ventricular function are indications for implantable cardioverter defibrilator (53). Patients who are hemodynamically unstable despite intensive medicamentous treatment may need intraaortic balloon counterpulsation pump, left ventricular assist devices, and in the most severe cases, heart transplantation (2). Patients with PPCM can develop heart failure in subsequent pregnancies.

## New Treatment Strategies

Data from researches related to the pathogenesis of PPCM were the basis for further investigations in the field of the treatment of PPCM.

### Pentoxifylline

*Pentoxifylline*, an inhibitor of TNFα, was evaluated in patients with PPCM. Significantly higher levels of CRP, IL6, TNFα were reported in patients with PPCM than in healthy controls having suggested that inflammation could be a cause of PPCM (24).

Sliwa et al. have studied the effects of pentoxifylline on outcome in patients with PPCM (9) and found that patients who received pentoxifylline as an addition to standard therapy had better outcome than those who were treated only with standard therapy. The role of pentoxifylline in the treatment of PPCM requires further evaluation.

### Bromocriptine

New researches by Hilfiker-Kleiner et al. suggested that oxidative stress and the cleavage of PRL may play a central role in the pathogenesis of PPCM (25). Based on these data, one pilot study in South Africa evaluated the effects of bromocriptine in patients with PPCM (54). The results of this study showed that patients who received bromocriptine in addition to standard therapy improved LVEF and clinical outcome. This suggests that bromocriptine could be used as a new specific therapy for PPCM. However, large randomized trials are needed before it can be used for the treatment of PPCM in clinical practice.

## Conclusion

Peripartum cardiomyopathy is a rare and very severe disease. Symptoms of PPCM can mimic symptoms of a normal pregnancy, which is the most common reason of delayed diagnosis. Delayed diagnosis almost always leads to severe forms of PPCM. Thus, attention should be paid on early diagnosis and treatment, which are crucial for better outcome. We should always have in mind the fact that the woman without previous heart disease can develop heart failure. Therefore, increased awareness of PPCM as well as high level of suspicion are the most important steps in establishing the early diagnosis. Early symptoms such as dyspnea, fatigue, leg edema, and malaise, which are often misinterpreted as physiological, should not be underestimated. Paroxysmal nocturnal dyspnea, nocturnal cough, and chest pain should raise suspicion of heart failure. PPCM is the diagnosis of exclusion. TTE is crucial not only as a diagnostic tool but also as a bedside non-invasive hemodynamic monitoring device. Treatment of PPCM is basically the same as for other non-ischemic cardiomyopathy, with the limitation concerning fetal development and wellbeing. The task for future studies is to find the cause of PPCM, and the specific biomarker of this disease, which would significantly contribute to early diagnosis and specific treatment of PPCM. In that context, studies that investigate inflammation, prolactin, and oxidative stress give promising results.

## Author Contributions

All aforementioned authors contributed significantly to the final design of manuscript.

## Conflict of Interest Statement

The authors declare that the research was conducted in the absence of any commercial or financial relationships that could be construed as a potential conflict of interest.
